# Sequence-Based Identification of Metronidazole-Resistant *Clostridioides difficile* Isolates

**DOI:** 10.3201/eid2811.220615

**Published:** 2022-11

**Authors:** Wiep Klaas Smits, Céline Harmanus, Ingrid M.J.G. Sanders, Lynn Bry, Grace A. Blackwell, Quinten R. Ducarmon, Eliane de Oliveira Ferreira, Ed J Kuijper

**Affiliations:** Centre for Microbial Cell Biology, Leiden, the Netherlands (W.K. Smits);; Leiden University Medical Center, Leiden, the Netherlands (W.K. Smits, C. Harmanus, I.M.J.G. Sanders, Q.R. Ducarmon, E.J. Kuijper);; Brigham & Women’s Hospital, Boston, Massachusetts, USA (L. Bry); Harvard Medical School, Boston (L. Bry);; European Bioinformatics Institute (EMBL-EBI), Hinxton, UK (G.A. Blackwell);; Wellcome Sanger Institute, Hinxton (G.A. Blackwell);; Universidade Federal do Rio de Janeiro, Rio de Janeiro, Brazil (E.O. Ferreira);; National Institute for Public Health and the Environment, Bilthoven, the Netherlands (E.J. Kuijper)

**Keywords:** *Clostridioides difficile*, Antimicrobial resistance, bacteria, enteric infections, metronidazole, plasmid, pCD-METRO, sequence analysis, the Netherlands, United States, South America, Brazil

## Abstract

The plasmid pCD-METRO confers metronidazole resistance in *Clostridioides difficile*. We showed high sequence similarity among pCD-METRO plasmids from different isolates and identified pCD-METRO and associated metronidazole-resistant isolates in clinical and veterinary reservoirs in the Americas. We recommend using PCR or genomic assays to detect pCD-METRO in metronidazole-resistant *C. difficile*.

*Clostridioides difficile* is a major cause of antibiotic-associated colitis (*1*). Antimicrobial drug–resistant infections are a global economic and healthcare burden (*2*). Resistance is generally low to commonly prescribed antimicrobial drugs used for primary *C. difficile* infections. However, high rates of metronidazole resistance have been observed for *C. difficile* isolates carrying the 7-kb plasmid pCD-METRO, in particular for isolates belonging to PCR ribotype (RT) 010 and RT020 (clade 1) and the epidemic strain RT027 (clade 2) (*3*) (Figure, panel A). This plasmid has been reported in *C. difficile* isolates from countries in Europe.

**Figure:**
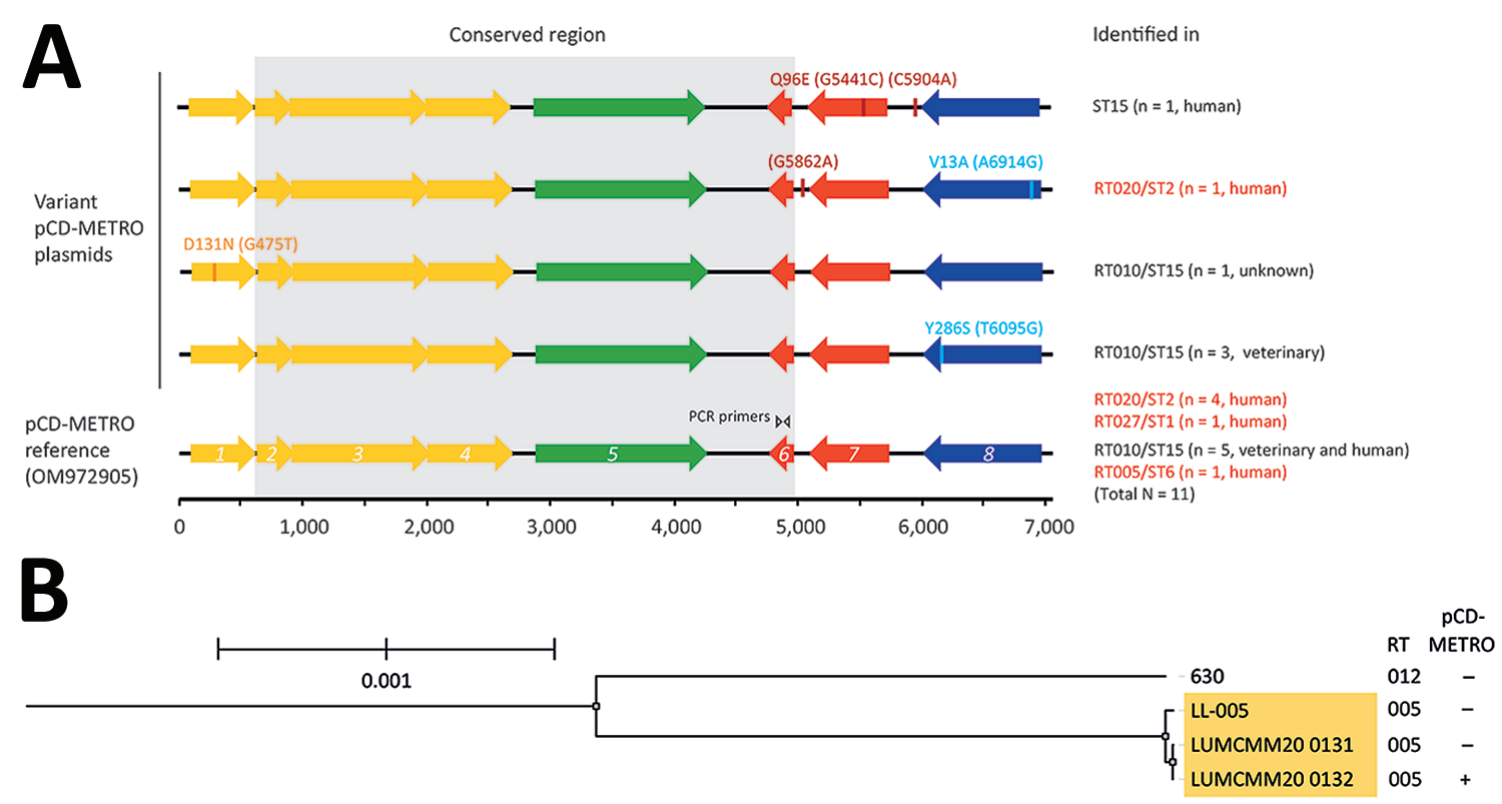
Comparison of pCD-METRO open reading frames and phylogenetic analysis in study of sequence-based identification of metronidazole-resistant *Clostridioides difficile* isolates. A) Linear maps compare the open reading frames (ORF)1–8 of the pCD-METRO reference sequence (identical to the RT005 plasmid) with variant pCD-METRO sequences, including the ST15 isolate from the United States (top). No ribotyping information was available for the ST15 isolate, but it should be noted that RT010 isolates belong to the same sequence type. Amino acid substitutions and nucleotide substitutions (in parentheses) are indicated above the ORFs. Colors indicate the location of putative mobilization genes (yellow), a replication gene (green), an integrase gene (blue), and genes encoding other functions (red) in the ORFs (*3*). The invariant regions are indicated by gray shading, and the binding location of the oBH1/2 primer set is shown in ORF6. The primer set is used for national sentinel surveillance and diagnostics of *C. difficile* infections in the Netherlands. Toxigenic RT/STs are indicated in red font and were all derived from symptomatic patients with *C. difficile* infections. Where available, the source (human/veterinary) is indicated. Isolate 1143 from Brazil was not included in this figure because no sequence information was available. B) Phylogenetic tree generated using IQ-TREE (*10*) and Roary (*11*) to show the relatedness between 2 RT005 patient isolates (LUMCMM20 0131 and LUMCMM20 0132) compared with the 2 reference strains LL-005 (RT005) and 630 (RT012). The tree is rooted on strain 630, and RT005 isolates are highlighted in yellow. Only the LUMCMM20 0132 isolate was positive for pCD-METRO. Scale bar indicates nucleotide substitutions per site. RT, ribotype; ST, sequence type.

## The Study

Since the discovery of pCD-METRO, we have implemented PCR that uses primers oBH-1 (5′-CCTCGTAGAATCCGGTGCAA-3′) and oBH-2 (5′-TATTTCCTTGCCGCTGAGGT-3′) for national sentinel surveillance and diagnostics of *C. difficile* infections in the Netherlands. The primers are specific for open reading frame (ORF) 6 of pCD-METRO (Figure, panel A). Since 2019, we have tested 3,257 isolates and identified 8 (0.25%) additional pCD-METRO–positive isolates; this percentage is consistent with previous findings (*3*). We have a total of 27 human and animal *C. difficile* isolates in our collection that are pCD-METRO–positive. Most of the isolates (22/27, 81%) belong to nontoxigenic PCR RT010, including isolate 1143 from Brazil. Isolate 1143 is one of 8 canine isolates that showed phenotypic resistance to metronidazole (MIC = 32 mg/L) by Etest on Brucella blood agar (BBA); the Etest was performed at the Universidade Federal do Rio de Janeiro in Brazil. The isolate from Brazil confirmed that pCD-METRO is present in *C. difficile* not only in Europe but also in South America. The 1143 isolate was not characterized further because it belonged to PCR RT010, in which pCD-METRO is most frequently observed. The high number of *C. difficile* RT010 isolates carrying pCD-METRO might be related to genomic background of the isolates (*4*) or sampling bias; a higher prevalence of metronidazole resistance has been observed among RT010 strains (*3*,*5*). Low-frequency horizontal gene transfer is more likely to occur after prolonged co-colonization of nontoxigenic *C. difficile* and pCD-METRO donor bacteria, and acquisition of the plasmid might occur from a source after metronidazole exposure. For example, dogs carry nontoxigenic *C. difficile* frequently and are often treated with metronidazole (*6*). The presence of pCD-METRO in toxigenic isolates might also be underestimated; antimicrobial susceptibility testing is not routinely performed, and plasmid carriage is not assessed, even when metronidazole treatment fails.

Among *C. difficile* isolates from the Netherlands, we identified a toxigenic pCD-METRO–positive isolate (LUMCMM20 0132, National Center for Biotechnology Information [NCBI] BioSample no. SAMN26573026) from a symptomatic patient with *C. difficile* infection. The isolate belonged to RT005, a ribotype not reported previously to carry pCD-METRO. RT005 accounts for ≈4% of *C. difficile* isolates in Europe (*7*) and shows a similar prevalence in the Netherlands. The patient did not respond to metronidazole treatment, and a metronidazole Etest on BBA, performed at Leiden University Medical Center, confirmed the isolate was metronidazole-resistant (MIC = 8 mg/L). In contrast, a plasmid-negative RT005 isolate obtained earlier from the same patient (LUMCMM20 0131, NCBI BioSample no. SAMN26573027) was metronidazole-susceptible (MIC = 0.125 mg/L), further suggesting acquired resistance after pCD-METRO acquisition. Illumina whole-genome sequencing (NCBI BioProject accession no. PRJNA814863) and analysis of draft genomes using Kbase (*8*) indicated LUMCMM20 0131 and LUMCMM20 0132 were highly homologous, had an average nucleotide identity (ANI) of >99.99%, and were categorized as sequence type 6, clade 1 (*9*). We performed phylogenomic analysis by using IQ-TREE (*10*) and Roary (*11*) to show the 2 patient isolates were distinct from the RT005 reference strain LL005 (ANI 99.91–99.92) and RT012 reference strain 630 (ANI 99.16–99.18) (Figure, panel B). Moreover, we identified only 1 single-nucleotide polymorphism (SNP) when we aligned reads from LUMCMM20 0132 in a reference assembly against the draft LUMCMM20 0131 genome (minimum coverage 10, minimum variant frequency 0.8). We revealed that differences in the 2 patient isolates were driven by pCD-METRO carriage in LUMCMM20 0132 in a pangenome analysis using Kbase (*8*). We identified the pCD-METRO contig in the draft genome by using a homology search, removed terminal repeats, and circularized the sequences by using Geneious R9.1 (https://www.geneious.com). The resulting plasmid sequence was 100% identical to the pCD-METRO reference sequence (GenBank accession no. OM972905) (Figure, Panel A), which likely explains the metronidazole-resistant phenotype.

Because the presence of pCD-METRO is rare, we identified pCD-METRO–positive isolates in public repositories. We queried a curated database of >661,000 assembled bacterial genomes (*12*) by using a compact bit-sliced signature index with a k-mer similarity threshold of 0.4. A total of 465 assemblies were returned, but only 1 *C. difficile* isolate had a close-hit of 0.99 k-mer similarity. The other hits had k-mer similarities of <0.49 and included different species. The *C. difficile* isolate containing a contig with sequence homology to pCD-METRO was V356 (NCBI BioSample no. SAMN08813897). V356 is a nontoxigenic sequence type 15 isolate cultured from an intensive care unit patient in the United States who was an asymptomatic *C. difficile* carrier; the isolate clustered with other nontoxigenic *C. difficile* genomes (*13*). The isolate was metronidazole-resistant (MIC = 16–24 mg/L) in an Etest on BBA medium (the test was performed at Brigham and Women’s Hospital at the time of identification). We assembled the whole-genome sequence of the isolate by using Kbase (*8*) and reconstructed the pCD-METRO plasmid from the draft genome sequence as described above. The plasmid had 2 SNPs compared with the pCD-METRO reference sequence: G5441C, resulting in a Q96E amino acid substitution in the ORF7 hydrolase protein, and C5904A upstream of ORF7 (Figure, panel A); other variants are described elsewhere (*3*). V356 extends the geographic range of pCD-METRO and associated plasmid-mediated metronidazole resistance to North America.

To facilitate homology-based identifications, we deposited a pCD-METRO sequence assembly (GenBank accession no. OM972905) for inclusion in databases of antimicrobial resistance and mobilization determinants, such as the Comprehensive Antibiotic Resistance Database (*14*) and PlasmidFinder (*15*). The deposited file also indicates the sequence variants described in this study.

## Conclusions

SNPs in pCD-METRO have been reported in ORF1, the ORF6–ORF7 intergenic region, ORF7, and ORF8, but not in the region that contains ORF2–6; major deletions or rearrangements in this plasmid have not been found. Thus, PCR-based approaches that detect conserved plasmid regions and genomic methods that examine pCD-METRO sequences can be used to identify pCD-METRO–containing *C. difficile* isolates. Of note, all isolates that carried pCD-METRO were confirmed to be metronidazole-resistant (MIC >2 mg/L) in susceptibility tests. Whereas the sequences responsible for metronidazole resistance in pCD-METRO have not yet been identified, we show that the presence of pCD-METRO in *C. difficile* predicts metronidazole resistance. We suggest using the invariant ORF2–6 region for PCR-based detection of pCD-METRO. 

We found pCD-METRO in a metronidazole-resistant toxigenic RT005 isolate from the Netherlands and also identified pCD-METRO–associated metronidazole resistance in *C. difficile* isolates from North and South America. We recommend using sequence-based molecular approaches to detect pCD-METRO for plasmid-mediated metronidazole-resistant *C. difficile*.
